# Primary Segmental Volvulus Mimicking Ileal Atresia

**Published:** 2013-01-01

**Authors:** Raghu Shankar, Sadashiva Rao, Kishan B Shetty

**Affiliations:** * Department of Pediatric Surgery, Justice K.S.Hegde Medical College and Hospital, K.S.Hegde Medical Academy, NIITE University, Mangalore, Karnataka, India.; 1 Department of Surgery, Kasturba Medical College, Mangalore, Manipal University, Karnataka, India

**Keywords:** Primary segmental volvulus, Neonate, Ileal atresia

## Abstract

Neonatal intestinal volvulus in the absence of malrotation is a rare occurrence and rarer still is the intestinal volvulus in absence of any other predisposing factors. Primary segmental volvulus in neonates is very rare entity, which can have catastrophic outcome if not intervened at appropriate time. We report two such cases, which were preoperatively diagnosed as ileal atresia and intraoperatively revealed to be primary segmental volvulus of the ileum.

## INTRODUCTION

Most cases of volvulus in the children, including the newborns, involve the midgut and are secondary to malrotation. Other reported causes of small bowel volvulus in children are congenital bands, post-operative adhesions, duplication cyst, meconium plug, meckel’s diverticulum, internal herniation, and ventriculoperitoneal shunt [1-4]. Primary segmental volvulus (PSV) is the torsion of a loop of bowel around the axis of its own mesentery in the absence of any other predisposing abnormalities. PSV is usually complicated by ischemic necrosis of the involved bowel necessitating resection. Herein, we present 2 cases of PSV in newborns. Both were thought to be cases of intestinal atresia, until per-operative findings revealed otherwise.

## CASE SERIES

**Case 1:** A full term, 3-day-old neonate, weighing 2.6kg, was referred with abdominal distension since birth and bilious nasogastric aspirates since day one of life. Antenatal scan had revealed no abnormality. The neonate had passed meconium on first day of life, and continued to pass good quantity of meconium subsequently. On examination, neonate was active with no clinical evidence of sepsis. Nasogastric aspirate was bilious (20-30ml/day). Abdomen was uniformly distended with no visible peristalsis. Plain abdominal radiograph was suggestive of neonatal intestinal obstruction. A diagnosis of ileal atresia was made and the neonate underwent exploratory laparotomy on 4th day of life. Distal ileal volvulus was encountered with gangrene of the entire 20 cm of the involved ileum (Fig. 1). The distal end of the gangrene was extending to the ileocecal junction. There was no evidence of malrotation, intestinal atresia, bands, duplication cyst or any other obvious pathology responsible for the volvulus. Resection of the gangrenous segment with the cecum, and an ileo-ascending colon anastomosis was carried out. The neonate passed stools on the 4th postoperative day, and oral sips were started. Anastomotic leak was noticed from the drain site on 6th postoperative day. This was managed conservatively as the leak was minor. The neonate continued to be fed orally as there was no distal obstruction. The leak healed by 14th postoperative day and the neonate was discharged on full feeds. In the follow-up visit (8 months), baby had gained weight and was thriving. 

**Figure:**
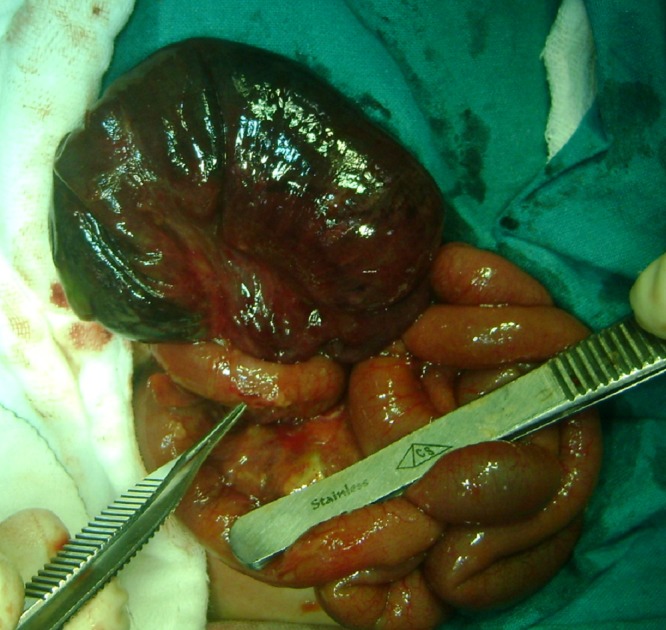
Figure 1: PSV in case 1.

**Case 2:** A preterm (33-34 weeks), 1.8 kg neonate was referred with abdominal distension and high nasogastric aspirates (40-50ml, bilious/day) on day two of life. Antenatal scan done 4 days prior to delivery had shown dilated bowel loops with multiple fluid levels. The neonate had passed meconium on first day of life. On examination, abdomen was mildly distended, but otherwise soft and non-tender. Plain abdominal radiograph was suggestive of small bowel obstruction. On laparotomy, proximal ileal volvulus with gangrene of the 15 cm of bowel was seen (Fig. 2). No obvious lesion leading to volvulus was found. Resection of the segment with anastomosis was done. The neonate had an anastomotic leak noted on 5th postoperative day. Reoperation with closure of the leak site was done and child recovered without any further problems. Baby had gained adequate weight in the follow up (18 months).

**Figure:**
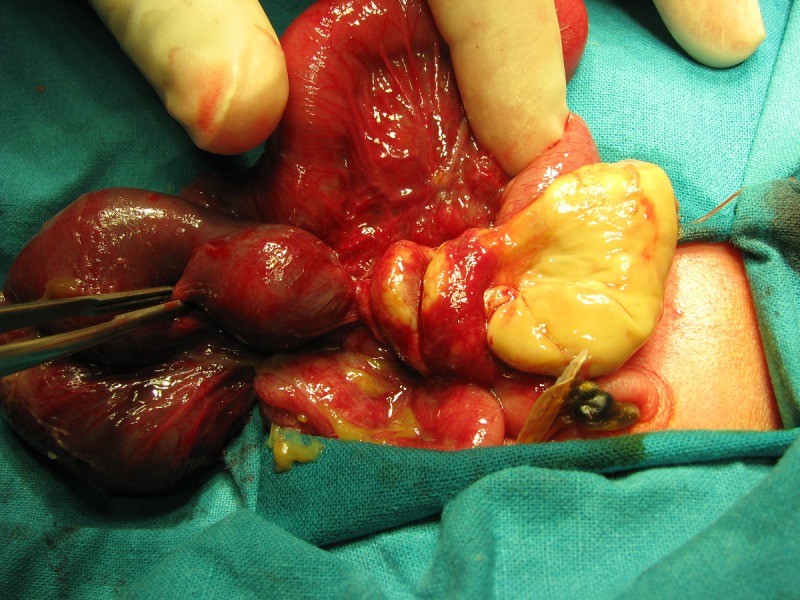
Figure 2: PSV in the second case.

## DISCUSSION

Most cases of intestinal volvulus in children are associated with malrotation. Some studies suggest that intestinal volvulus without malrotation occurs in 19-26% of the small bowel volvulus in children [1, 5]. However, most of these have other predisposing causes like meckel’s diverticulum, postoperative adhesions, duplication cyst, meconium plug or internal herniation. PSV of the ileum occurring in a neonate is extremely rare. There are only few reports of this entity. Majority of the previous reports describe it in preterm neonates [1, 3, 6], however, we had a full term neonate with this condition. Like other intrauterine mesenteric vascular insults, PSV can also cause vascular insufficiency and lead to ileal atresia. It has been postulated that if these children were to be born at term they would have presented with ileal atresia [6, 7].

The etiology of PSV remains unknown. Stasis of the bowel content, long, narrow, band-like mesentery; rapid changes in the intra-abdominal pressure; and hyper-peristalsis have been proposed as possible mechanisms [8, 9]. In both of our cases mesentery was wide. 

Exact preoperative diagnosis cannot be made with certainty. Plain X-ray abdomen would show features of intestinal obstruction. Reports by Kitano et al and Jung et al mention barium enema findings which may be seen in some cases and could aid in the diagnosis. The contrast should go across the ileocecal junction into the terminal ileum. The features seen could be a ‘bird-beak’ appearance indicating a knot in the distal ileum or abnormal coiling of the terminal ileum [1, 6]. However, both the author groups had noted these in retrospect. The most common preoperative diagnosis would be of intestinal atresia or stenosis. Once these diagnoses are made, immediate laparotomy may not be required in the odd hours as long as the proximal bowel is kept decompressed adequately. Hence, usually there is a delay in intervention. Compared to volvulus with malrotation, ischemic changes of PSV are thought to progress rapidly because the colon which otherwise plays the role of a cushion is not involved [8]. A fixed cecum results in a very tight volvulus with rapid ischemia and irreversible necrosis compared to mobile cecum which results in a less tight volvulus and less severe ischemia and delayed necrosis [10]. This is the reason for gangrene which is seen in all the cases of PSV, which necessitated resection of the involved segment. In a report by Kitano et al, they found 90% of the PSV had ischemic changes and 40% had perforation when compared to only 15% bowel strangulation in cases of volvulus with malrotation [1]. However, midgut volvulus with gangrene of the involved bowel would be more catastrophic leading to perforation or sometimes loss of the entire small bowel when compared to PSV, where a lesser length of the bowel is involved.

To conclude, PSV is very rare entity; the prognosis can be improved by keeping a high index of suspicion and early intervention. Resection of volvoluted bowel is always necessary but length of the remaining bowel is usually sufficient to allow normal development.

## Footnotes

**Source of Support:** Nil

**Conflict of Interest:** None

## References

[1] ( 1995). Kitano Y, Hashizume K, Okhura M. Segmental small bowel volvulus not associated with malrotation in childhood. Pediatr Surg Int.

[2] ( 1995). Maung M, Saing H. Intestinal volvulus: an experience in a developing country. J Pediatr Surg.

[3] ( 2003). Chen Y, Tseng SH, Lai HS, Chen WJ. Primary volvulus of the ileum in a preterm infant. J Formos Med Assoc.

[4] ( 2000). Ameh EA, Nmadu PT. Intestinal volvulus: aetiology, morbidity, and mortality in Nigerian children. Pediatr Surg Int.

[5] ( 1990). Lister J. Malrotation and volvulus of the intestine. In Lister J, Irving IM, Rickham PP, editors. Neonatal Surgery.

[6] ( 2011). Jung E, Choi SO, Park WH. Primary segmental volvulus of the ileum mimicking meconium plug syndrome. J Korean Surg Soc.

[7] ( 1997). Hong J. Ileal atresia due to intrauterine intussusception. J Korean Surg Soc.

[8] ( 1978). Kurashige T, Matsuyama S. Primary volvulus of the small intestine in infants. Jpn J Surg.

[9] ( 1991). Usmani SS, Kenigsberg K. Intrauterine volvulus without malrotation. J Pediatr Surg.

[10] ( 1989). Vergnes Boissinot F, Pontailler JR. Primary volvulus of the small intestine without malrotation. Apropos of 7 cases. Ann Pediatr.

